# Doctors and Newspaper Advertising

**Published:** 1902-04

**Authors:** 


### EDITORIAL NOTES AND COMMENTS.

The Business office of THE JOURNAL-RECORD is No. 64 Marietta Street.

The Editorial office is 318-319-320 The Prudential.

Address all Business communications to Dr. M. B. Hutchins, Mgr.

Make remittances payable to THE ATLANTA JOURNAL-RECORD OF MEDICINE.

On matters pertaining to the Editorial and Original Communications address Dr. Bernard Wolff, Atlanta.

Reprints of original articles will be furnished at cost price. Requests for the same should always be made *on the manuscript*.

We will present, post-paid, on request, to each contributor of an original article, twenty (20) marked copies of THE JOURNAL-RECORD containing such article.

### DOCTORS AND NEWSPAPER ADVERTISING.

A JOURNAL for advertisers contains the following editorial paragraph :

Physicians never were able to advance one good reason for refusing to advertise in newspapers and magazines, the one plea they use, that quacks pay for publicity, is illogical. By parity of reasoning, the honest merchant or manufacturer should decline to buy newspaper space because there are unscrupulous merchants and manufacturers who also advertise. The reputable physician, by his own confession, keeps out of print, leaving to the charlatan all the splendid benefit of publicity and permitting him to deceive the public at pleasure. Incidently reporters know that physicians are only too ready to give their names and address for publication in any case deserving of newspaper mention in which their professional services have been called into requisition.

It is astonishing that the "Little Schoolmaster," as this publication delights to call itself, cannot discern the motive that supplies good and sufficient reasons why physicians do not advertise in newspapers and magazines. This involves a fundamental principle and is that of simple honesty. It may be freely confessed that the lines are too rigidly drawn in places, but the essential spirit of the ethical injunction is at the same time correct and proper. There can be no legitimate objection to a card in a local paper stating the fact that Dr. So and So limits his practice to such and such a province of medicine, and that he may be found at a given address within certain hours. This is an unpretentious announcement for the information of the public, and contains no boastful assertion of superior knowledge or attainment. It is a practice that is still countenanced in some localities and was until recently in vogue in Atlanta. But it is easily understood that advertising must not proceed beyond this safe and unequivocal limit, for it marks the line of separation between the admissible and the inadmissible. Departure from the straight and narrow way leads on to quackery immediately and inevitably. The next advance must necessarily consist in claims to higher medical attainments, evidenced in multiplicity of diplomas, years of practice, glittering success in the treatment of diseases adjudged chronic or irremediable, special and secret methods *et id hoc omne genus*, the hall marks of the quack. If a doctor hitherto bearing an unimpeachable name for ethical observance announces in the daily papers that he has discovered a remedy for the cure of cancer, or Bright's disease, or dropsy, he has separated himself from the company of the elect and is at once identified, and very justly identified, with charlatans, because the distinction between regular and ethical methods and conduct and irregular and unethical is absolutely fixed and cannot be misunderstood or misinterpreted. Why not? Because no honest and ethical practitioner is willing to make claims which his own experience and the added experience of others prove cannot be substantiated. Individual variation to disease and reaction to therapeutics will forever render it impossible honestly to guarantee a cure except in certain surgical conditions, and even then it is unwise not to consider the unforeseen possibilities of error.

These are some of the reasons why physicians do not advertise in newspapers and magazines. They have no wares to sell and values to boast. They prefer to be honest than to have the splendid gains of the quack when these gains are acquired by fraud, misrepresentation and lying. So that after all it is a matter of simple honesty, which does not seem to appeal to the mind of the "Little Schoolmaster" as longer existing in this degenerate world.

